# Supplementary figures and images for: Deletion of IFT20 exclusively in the RPE ablates primary cilia and leads to retinal degeneration
Source: PLoS Biol. 2023 Dec 4;21(12):e3002402. doi: 10.1371/journal.pbio.3002402 (PMC10721183; doi:10.1371/journal.pbio.3002402)

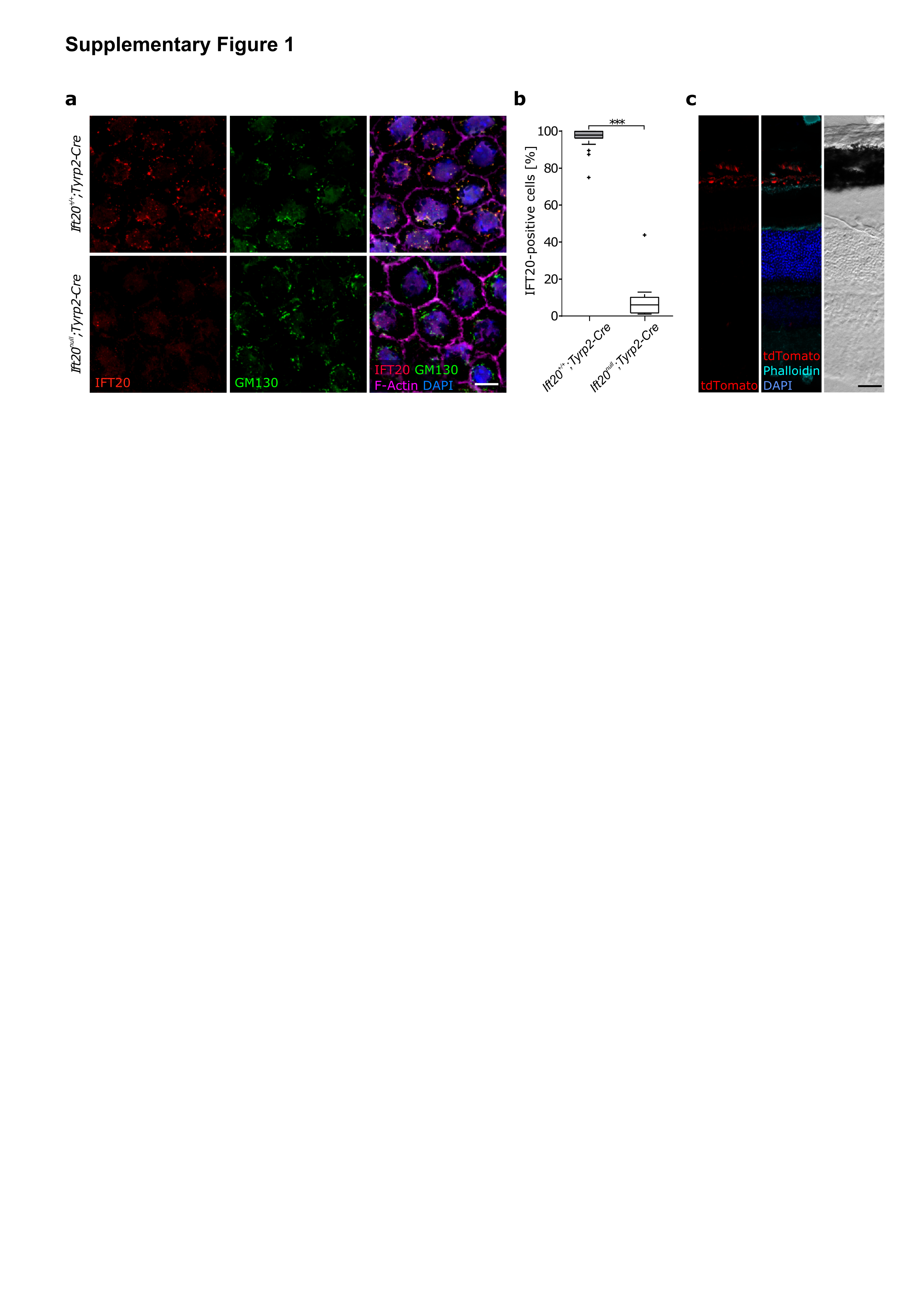

Supplement: S1 Fig — (a) Representative fluorescent images of E16.5 RPE flatmounts stained for IFT20 (red) and cis-Golgi matrix protein GM130 (green). Staining for F-Actin (magenta) was used to visualize the cytoskeleton, whereas DAPI was used to stain nuclear DNA. E16.5 Ift20null;Tyrp2-Cre RPE showed less IFT20 staining compared to controls. Scale bars: 10 μm. (b) Quantification of IFT20 positive cells in E16.5 RPE revealed that Ift20null;Tyrp2-Cre RPE showed near to no IFT20 staining (5.3% n = 4 (1,222 cells)) compared to control (98.5% n = 4 (1,236 cells)), confirming the knockout. Statistical analysis was performed using ROUT test (Q = 0.1%) before using unpaired t test (p < 0.001). Median: Ift20+/+;Tyrp2-Cre 98.5%, Ift20null;Tyrp2-Cre 5.3%. (c) Representative retina cross section of 1-month-old Ift20null;Tyrp2-Cre mouse crossed with a tdTomato reporter mouse. F-Actin staining visualized by Phalloidin staining (light blue) and DAPI for nuclei (blue). tdTomato expression (red) representing Cre activity was detectable only in RPE. Scale bar: 20 μm. Significance levels: >0.05 not significant (ns), <0.05*, <0.01**, <0.001***. Box plot: Box limits represent the first and third quartile, the central line shows the median and the whiskers indicate the 5th and 95th percentile. Numerical data can be found in S7 Table. (TIFF) [file pbio.3002402.s001.tiff]

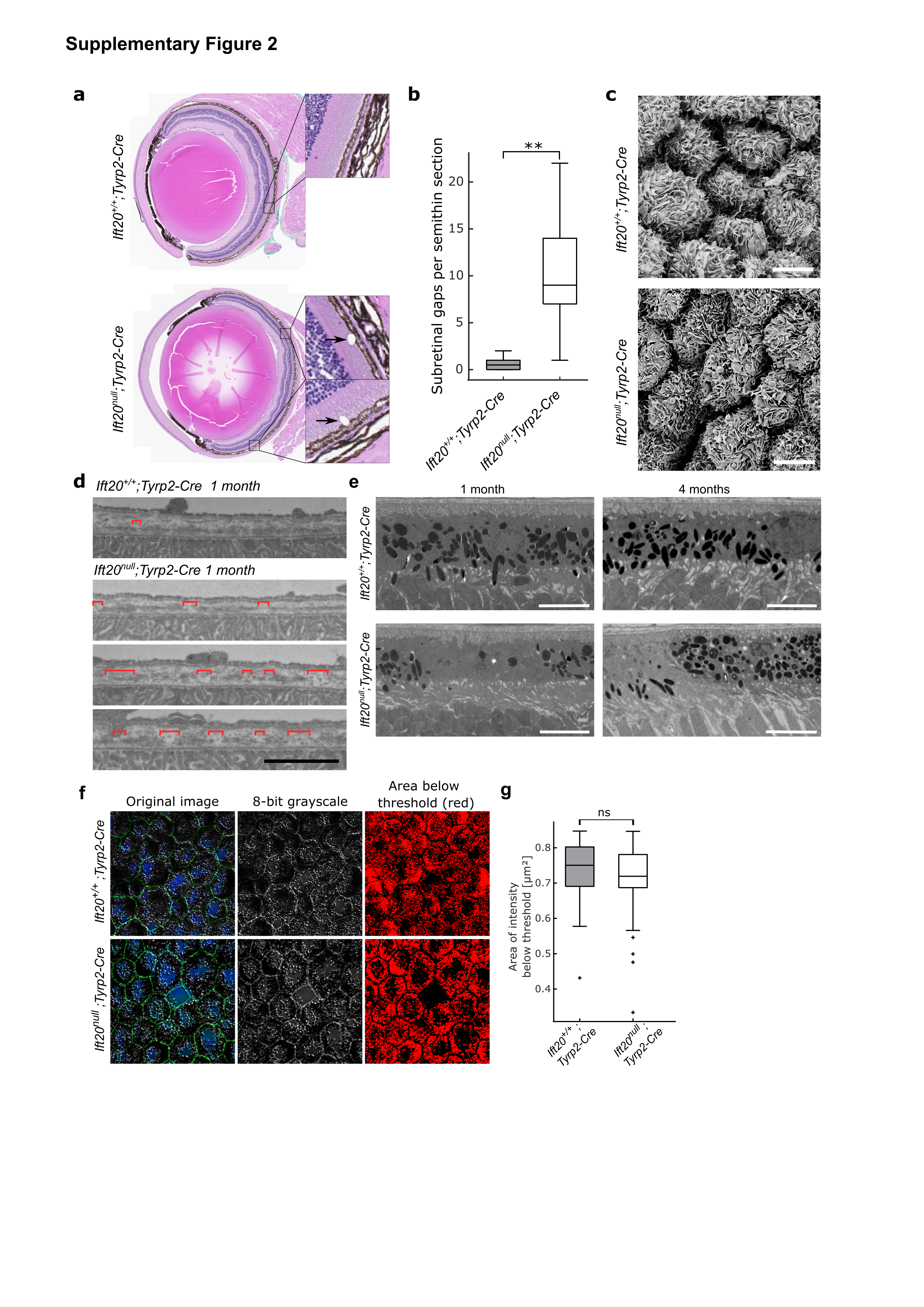

Supplement: S2 Fig — (a) HE-stained cross section through the mouse eye reveals subretinal gaps in the outer layers of Ift20null;Tyrp2-Cre retina compared to Ift20+/+;Tyrp2-Cre. (b) Quantification of subretinal gaps revealed a median of 0.5 gaps in Ift20+/+;Tyrp2-Cre mice and 9 gaps in Ift20null;Tyrp2-Cre mice per semithin section quantified (Ift20+/+;Tyrp2-Cre n = 9 sections, 4 eyes, 3 mice, Ift20null; Tyrp2-Cre n = 9 sections, 4 eyes, 3 mice). (c) Representative SEM images of P0 RPE flatmounts. No differences in microvilli morphology were observed between P0 Ift20null;Tyrp2-Cre and Ift20+/+Tyrp2-Cre RPE. Scale bar: 5 μm. (d) Representative TEM images of eye sections from 1-month-old mice showing a discontinuous Bruch’s membrane (red brackets) at Ift20null;Tyrp2-Cre RPE cells. Scale bar: 2 μm. (e) Representative TEM images of Ift20null;Tyrp2-Cre show abnormal pigmentation at 1 month and 4 months. Two pigmented RPE cells flank a cell almost completely devoid of melanosomes at 1 month. At 4 months of age, we observed an RPE cell that appeared devoid of melanosomes in the cell body, which was flanked by an RPE cell with an excessive accumulation of melanosomes. Scale bar = 10 μm. (f) Flatmount preparations from P0 mice imaged via differential interference contrast (DIC) overlayed with immunohistochemistry for cell nulcei (DAPI) and cell borders (ZO-1). Images were converted to 8-bit grayscale and masked to calculate pigmentation area. (g) Quantification of pigmentation in terms of area covered. No significant differences could be detected between control and mutant animals. Median: Ift20+/+;Tyrp2-Cre 7.5 μ2 area of intensity below threshold, Ift20null;Tyrp2-Cre 7.9 μ2 area of intensity below threshold. Ift20+/+;Tyrp2-Cre (n = 9 eyes (44 images)), Ift20null;Tyrp2-Cre (n = 6 eyes (27 images)). Significance levels: >0.05 not significant (ns), <0.05*, <0.01**, <0.001***. Box plots: Box limits represent the first and third quartile, the central line shows the median and the whiskers indicate t [file pbio.3002402.s002.tiff]

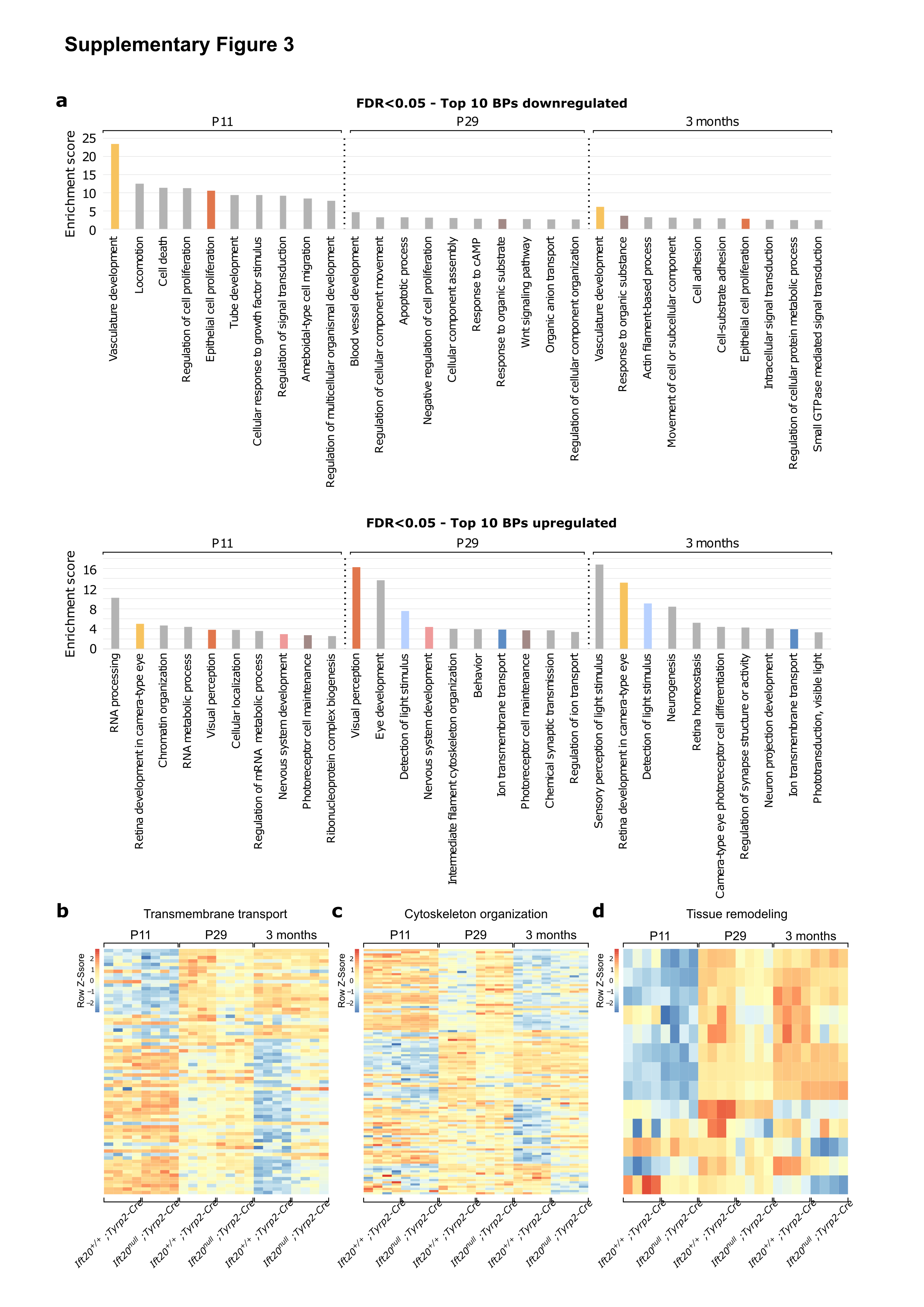

Supplement: S3 Fig — (a) Top: Identification of the biological processes underlying the effect of the deletion of Ift20 via gene ontology analysis. The top 10 down-regulated significant biological processes (BPs) in Ift20null;Tyrp2-Cre vs. Ift20+/+;Tyrp2-Cre are shown. The Enrichment score for each BP cluster is plotted on the y-axis. Bottom: Identification of the biological processes underlying the effect of the deletion of Ift20 via gene ontology analysis. The top 10 up-regulated significant biological processes (BPs) in Ift20null;Tyrp2-Cre vs. Ift20+/+;Tyrp2-Cre are shown. The Enrichment score for each BP cluster is plotted on the y-axis. (b–d) Heatmaps showing DEGs associated with transmembrane transport (b), cytoskeleton organization (c), and tissue remodeling (d). Data has been deposited in the GEO database (GSE144724). (TIFF) [file pbio.3002402.s003.tiff]

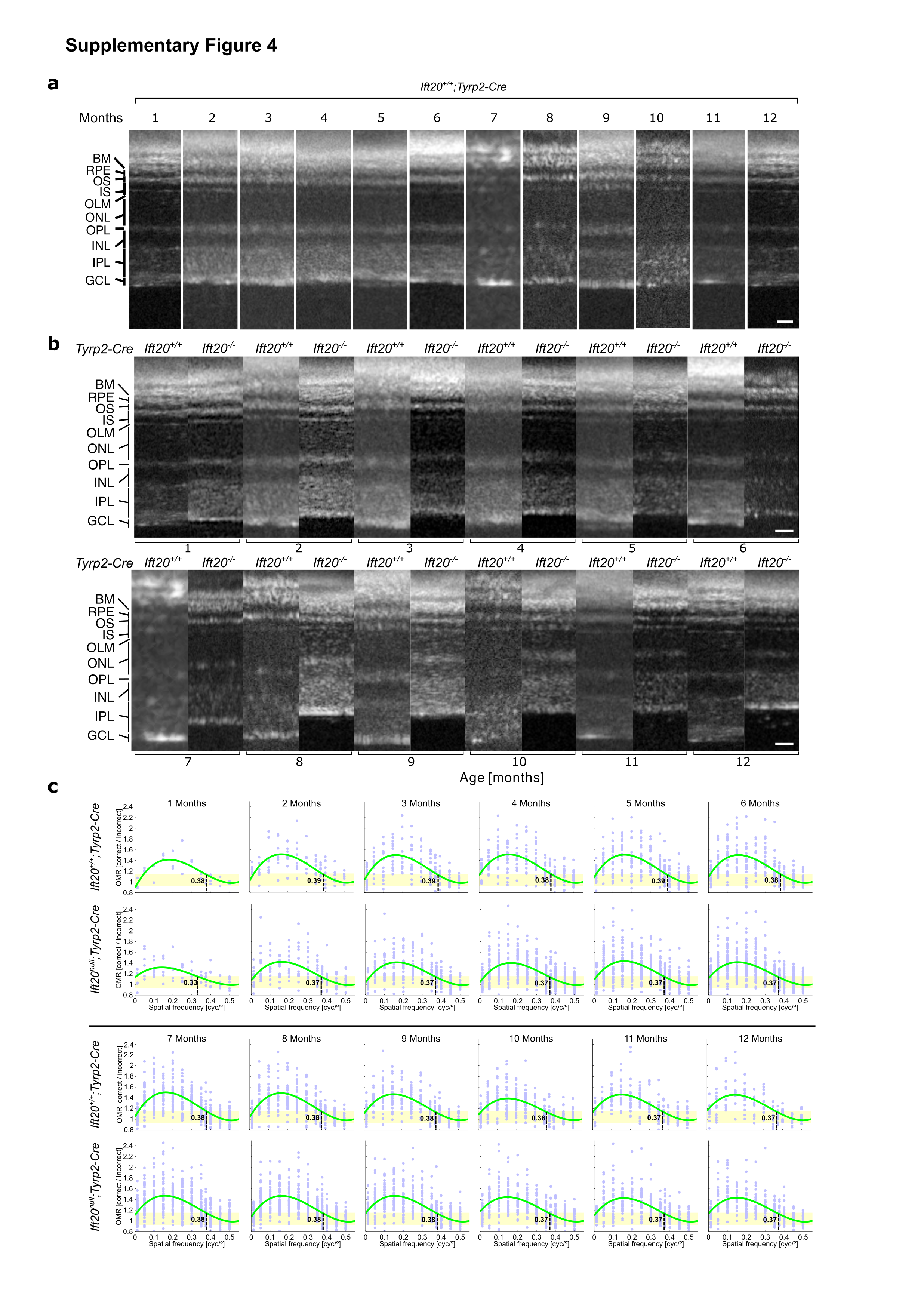

Supplement: S4 Fig — (a) Representative images of in vivo OCT scans of Ift20+/+;Tyrp2-Cre mice from 1 to 12 months. No overall change in could be seen over time. Scale bar: 25 μm. (b) Representative Images of in vivo OCT scans of Ift20null;Tyrp2-Cre mice compared to Ift20+/+;Tyrp2-Cre at monthly intervals from 1 to 12 months. Scale bar: 25 μm. (c) Optomotor response curves of Ift20+/+;Tyrp2-Cre and Ift20null;Tyrp2-Cre mice. Single measurements (purple dots) show a normal flicker. Visual acuity thresholds remained stable at around 0.38 cyc/° (dashed line, spatial frequency threshold) in both, Ift20+/+;Tyrp2-Cre and Ift20null;Tyrp2-Cre mice over the time of 12 months. For all experiments n > 4 animals. Numerical data can be found in S7 Table. (TIFF) [file pbio.3002402.s004.tiff]

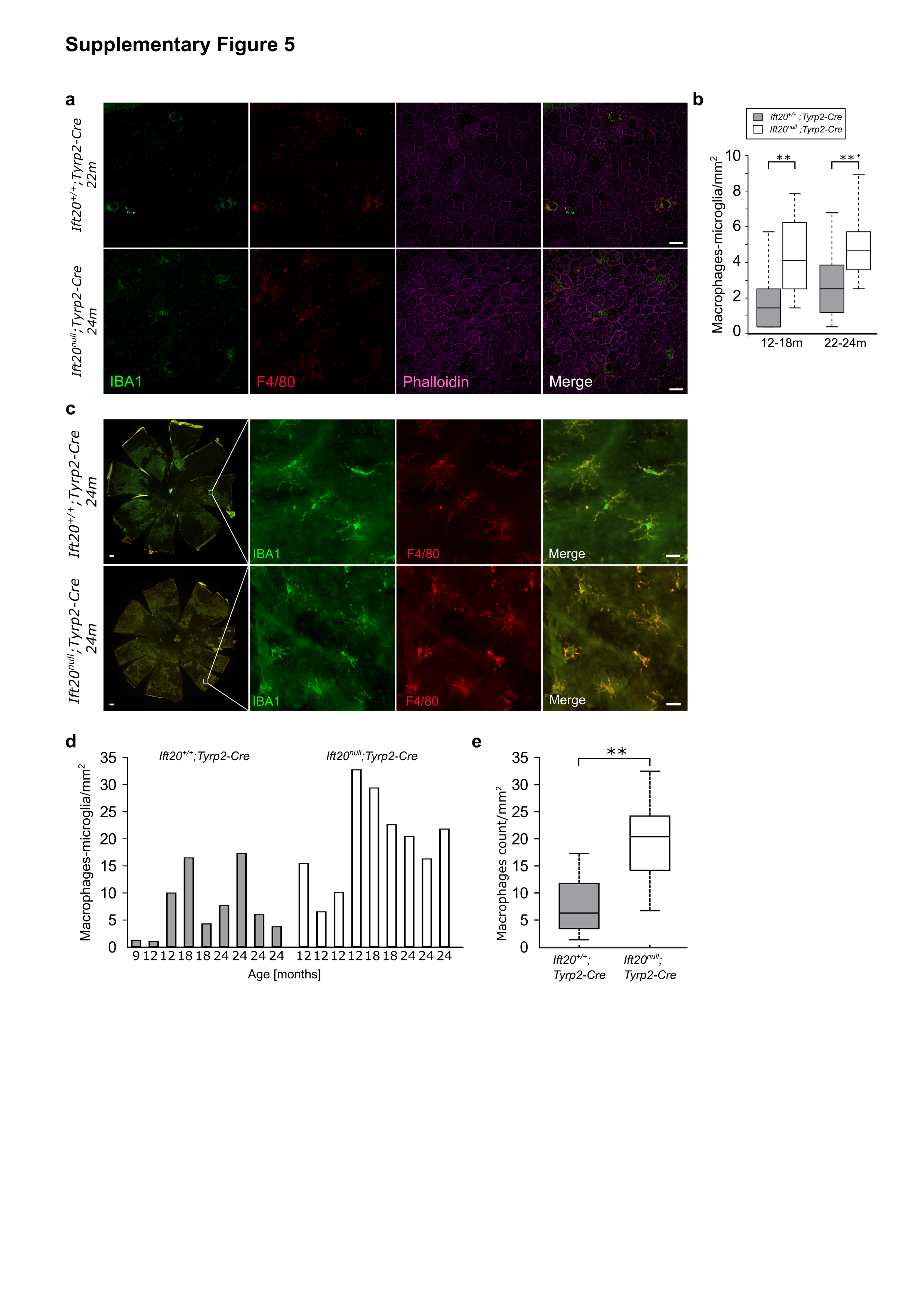

Supplement: S5 Fig — (a) Representative images of RPE flatmounts of Ift20+/+;Tyrp2-Cre and Ift20null;Tyrp2-Cre at 22 and 24 months of age. Macrophages were stained by markers IBA1 (green) and F4/80 (red). (b) Quantification of macrophage numbers per mm2 separated by age. Median: 12–18m Ift20+/+;Tyrp2-Cre 1 macrophage/mm2, Ift20null;Tyrp2-Cre 4.18 macrophages/mm2. 22–24m Ift20+/+;Tyrp2-Cre 2.5 macrophages/mm2, Ift20null;Tyrp2-Cre 4.31 macrophages/mm2. Statistical analysis was performed using the unpaired two-tailed t test. 12–18m Ift20+/+;Tyrp2-Cre n = 6 eyes 7 images, Ift20null;Tyrp2-Cre n = 4 eyes 7 images. 22–24m Ift20+/+;Tyrp2-Cre n = 4 eyes 7 images, Ift20null;Tyrp2-Cre n = 6 eyes 7 images. (c–e) Combining all ages revealed a significant difference in macrophage number between Ift20+/+;Tyrp2-Cre and Ift20null;Tyrp2-Cre. (c) Representative images of entire RPE flatmount of Ift20+/+;Tyrp2-Cre and Ift20null;Tyrp2-Cre at 24 months of age. Macrophages were stained by markers IBA1 (green) and F4/80 (red). Median: Ift20+/+;Tyrp2-Cre 7.8 macrophages/mm2, Ift20null;Tyrp2-Cre 20.9 macrophages/mm2. Statistical analysis was performed using the unpaired two-tailed t test. Ift20+/+;Tyrp2-Cre n = 9 flat mounts, Ift20null;Tyrp2-Cre n = 9 flat mounts. Scale bar: 20 μm. Significance levels: >0.05 not significant (ns), <0.05 *, <0.01 **, <0.001 ***. Box plots: Box limits represent the first and third quartile, the central line shows the median and the whiskers indicate the 5th and 95th percentile. Numerical data can be found in S7 Table. (TIFF) [file pbio.3002402.s005.tiff]
