# Supplementary material for: Deletion of IFT20 exclusively in the RPE ablates primary cilia and leads to retinal degeneration
Source: PLoS Biol. 2023 Dec 4;21(12):e3002402. doi: 10.1371/journal.pbio.3002402 (PMC10721183; doi:10.1371/journal.pbio.3002402)
Supplement: Attachment — Submitted filename: Kretschmer et al. IFT20 Response to reviewers comments.docx [file pbio.3002402.s013.docx]

**Response to reviewers comments**

**Reviewer 1:**

The manuscript by Kretschmer et al is an interesting study regarding the importance of IFT20 in RPE cells to retinal health. The RPE specific knockout of IFT20, a gene known to be crucial for the maintenance of primary cilia, results in a slow, progressive retinal degeneration associated with defects in RPE cells. The data presented are extensive and mostly of high quality. This study does a nice job in quantification of observed phenotypes. The major fault of the paper is that the phenotype induced by knockout of IFT20 occurs throughout the adult life of the mouse (very slow), when few, if any, RPE cells have a primary cilia. How could the effects of IFT20 knockout be exerted through ciliary defects if RPE do not have cilia to begin with? This point is essentially not addressed. Further, the potential non-ciliary functions of IFT20 are not mentioned or explored. This study should be revised to address these issues and present the data about the phenotype associated with IFT20 deletion from the RPE cells, rather than presume that the effects are through cilia ablation.

*Response: We appreciate time and effort that this reviewer took to review our manuscript and are grateful that they acknowledged the quality of the data. Previous reports by ourselves and others had shown that cilia in the RPE are required for development and maturation of the RPE, after which the cilium retracts. In this report we wanted to highlight that this has long lasting consequences, ultimately leading to retinal degeneration. We have now expanded this point in the discussion to make this clearer. We have also updated the discussion to explore the possibility of non-ciliary functions of IFT20. In response to the editor's request, we have thoroughly reorganized the data and figures in this manuscript. We are optimistic that the adoption of the short report format will enhance the conciseness of the story.*

Concerns and comments:

1. Figure 1h, please indicate the age of the mouse analyzed either in the results section or in the figure legend.

*Response: Thank you for spotting this. This information has now been added to the figure legend.*

2. Figure 2e, nowhere in the manuscript is it described how melanin concentration was obtained. Please describe in the results, methods and/or figure legend. Also, a whole retina measurement of melanin should be performed to consider the possibility that there is less melanin to begin with in the IFT20 KO RPE. The image from Fig. 1e suggests this may be the case. Later in the manuscript, the variation in melanosomes is demonstrated (Supplement Fig 2) which bolsters the idea that there could be a reduction in total melanin causing this effect. A formal quantification of total melanin should be performed.

*Response: We have now updated the methods section to describe the retinal adhesion assay and determination of melanin concentration in more detail. Regrettably, the reshape software lacks the capability to measure pigmentation, thereby limiting high content image analysis of entire flatmounts at a single cell resolution. Nevertheless, using differential interference contrast (DIC) microscopy, we successfully assessed pigmentation; however, no significant disparity was observed between the control and mutant groups. As pointed out by the reviewer, occasional instances of abnormal pigmentation were observed in the mutant cells during transmission electron microscopy (TEM), indicating sporadic and spontaneous occurrences.*

3. Figure 2f, Is one of these the KO? They are both labeled IFT20+/+;Tyrp2-Cre. In these phagocytosis assays, how do you differentiate between POS that are phagocytosed with POS that are just adhered to the RPE? In Fig. 2e, you claim that the retina adheres more gently to the RPE which is consistent with you measuring a reduced binding of POS to the RPE in your phagocytosis assays.

*Response: Yes, the author is correct one assay was from control cultures the other from mutant cultures. We have now corrected the typo.*

*Unfortunately, we did not have enough eyes/independent wells to be able to look at both binding and internalization in these assays therefore we cannot distinguish between bound and internalized POS. However, the binding of POS preceding their internalization and retinal adhesion are two independant processes. Indeed, it has been shown previously that the integrin receptor alphavbeta5 is implicated in both processes but with different ligands and timings. POS binding is synchronized by the receptor-ligand duo alphvbeta5--mfg-e8 and has a peak of activity 1.5-2 hours after light onset (Nandrot et al, 2004 J Exp Med; Nandrot et al, 2007 PNAS). POS binding relies on short microvilli at the RPE apical surface encompassing aged POS tips exposing phosphatidylserines indicating which part of the cell needs to be eliminated (Ruggiero et al, 2012 PNAS). On the other hand, retinal adhesion involves long RPE microvilli and the whole length of photoreceptor outer segments. Its activity peak occurs 3.5 hours afters light onset and is lead by alphavbeta5 integrin receptors but with a still unknown ligand (Nandrot et al, 2006 Am J Physiol Cell Physiol; Nandrot et al, 2007 PNAS). Hence, both functions being independent, decreased retinal adhesion is not automatically associated with decreased POS binding and thus internalization but it might be the case as suggested by our results.*

*We have now added this aspect to the discussion.*

4. Figure 4, when looking at the engulfment of POS by the RPE, what time of day were the mice sacrificed? Ideally, this should be noted in the methods/figure legend and the controls must have been sacrificed at the same time as the experimental mice.

*Response: We made a point to be as consistent as possible and all animals were scarified at the same time of day. Namely three hours after light onset. This has now been added to the methods.*

5. Figure 4h, The term "microvilli depth" should not be used because it implies that you are measuring the length of microvilli. In fact, you have measured the space between the RPE apical surface and the tips of the photoreceptor outer segments. Perhaps it could be called "RPE to POS distance"

*Response: Thank you for the suggestion. We agree with the reviewer and have made these changes.*

6. Line 220 - The title of this section says "single-cell analysis" which is a commonly used term for analysis of single-cell sequencing data. I suggest changing this title to avoid confusion.

*Response: We have changed this term to ‘single-cell-resolution analysis’ as it was originally described in paper that developed the method (Ortolan et al., PNAS, 2022).*

7. Line 239 - 240 - The statement that RPE cell patterning defects could not be due to cell loss does not make sense. The cell area gets very slightly bigger. Does the retina and/or RPE layer get proportionally bigger as compared to WT? **Probably not, but the authors could measure.** If the RPE layer/retina stays the same size, then the number of RPE cells must be dropping as compared to WT. The larger individual RPE cells, and therefore lower RPE cell density, implies that some have died, leaving room for individual cell growth. Looking at the RPE cell size/density at a much later timepoint would help to answer this question. Because the reduction in RPE cell size is so small, the putative loss of RPE cells would also have to be small during the studied timeframe to P90. Given that caspase activation would happen on the order of minutes, it would likely be impossible to catch these rare events happening at any given time. Therefore, the authors cannot rule out that RPE cell
death is contributing to cellular morphology changes. The caspase data should be removed from the paper.

*Response: To examine this somewhat further we have reanalyzed the cell size data. We have now included the cell area data binned at intervals of 50μm^2^ (Figure 2c). Interestingly the binned profiles look remarkably similar between the control and the mutant across all three ages examined. Suggesting that there are no mayor differences in cell size ratios, despite a slight increase in total cell size in the two older categories. In future studies we are keen to explore these changes in much older animals, particularly since this is when we see increased expression of macrophage and microglia markers, which might be a response to cell loss.*

*We agree with the reviewer that the caspase data is confusing, and it has now been deleted.*

8. The figure legend for Figure 6 is swapped with Figure 5.

*Response: Upon the editors request we have completely restructured the data and figures in this manuscript. We have taken great care to make sure all the Figure numbers are correct in the new version.*

9. Line 255 - The text should reference Figure 7b here.

*Response: Since this statement ‘across all ages more genes were down-regulated than up-regulated compared to controls‘ refers to the data in both 7a and 7b (now Figure 3a, b). We have added both.*

10. Line 745 - Figure 8g is microscopy images, not western blot as described in the legend.

*Response: Upon the editor’s request, this data has now been removed.*

11. Line 346 - The correct point brought up in Discussion that ciliary proteins may have non-ciliary roles is essentially not addressed in this paper. The disease observed by the authors with IFT20 KO RPE is very slow and occurs over a timeframe when the vast majority of RPE do not have cilia. How could a ciliary defect cause this long-term pathology in cells that lost their cilia during development? Specific non-ciliary roles of IFT20 are not discussed, despite a seemingly abundance of literature on the topic (see PMID: 33072760 for recent review). The concept that IFT20 is doing something important in fully developed RPE that lack cilia should be strongly considered/discussed/tested. This concern is highlighted by the fact that the title of the paper starts with "Ablation of primary cilia…" when it should begin with "Knockout of IFT20…".

*Response: We have expanded this point in the discussion and make it clear that non-ciliary functions of IFT20 might also be playing a role here. It is plausible that we are witnessing a compounded effect involving both developmental defects leading to immature/pathogenic RPE and subsequent effects resulting from the loss of IFT20 in adulthood. This effect may arise from the remaining few ciliated cells or from non-ciliary functions of IFT20. To investigate this matter further, we intend to employ inducible IFT20-RPE Cre knockout models in future studies.*

*Additionally, we have emphasized that IFT20 remains one of the most appropriate models for investigating the implications of ciliary loss. This is because the loss of other intraflagellar transport proteins (such as IFT88) still allows for the presence of a ciliary stump above the transition zone. Given that IFT20 is essential for ciliary vesicle trafficking to the basal body, its loss generates one of the most comprehensive models for ciliary ablation.*

*We have also revised the title to reflect the loss of IFT20. However, we find it crucial to emphasize the loss of cilia for readers who may not be familiar with ciliary biology in order to ensure comprehensibility of the article.*

12. The data described and shown in Figures 7 and 8 are in regard to alleged RPE expressed genes. However, many of them are not genes expressed by the RPE. The majority of the genes shown in Figure 8d are photoreceptor specific genes, including rhodopsin, transducin, recoverin and cone opsin. Unfortunately, this confounds the interpretation of their global gene expression analysis and makes these data have little value. Single cell RNA sequencing would have been a preferred method for this analysis. The fact that the photoreceptor specific genes drastically go up at later time points indicates that more photoreceptor cells were contaminating the RPE cell isolation at these timepoints. How could it be possible to determine what fraction of other gene changes are due to this contamination? Unless this could be addressed, these two figures should be moved to the supplement with a disclaimer that their interpretation is confounded by contamination.

*Response: In accordance with the reviewer's suggestion, the majority of this data has been relocated to the supplements. We have streamlined the analysis of this data to avoid overinterpretation of the results. Furthermore, we have acknowledged the limitations of this approach compared to single-cell RNA sequencing and recommend a focus on RPE-specific genes for further analysis.*

**Reviewer 2:**

This manuscript by Kretschmer and colleagues investigates whether ciliary defects in retinal RPE cells could play a pathological role in retinal dystrophy by creating and characterizing a mouse model that disrupts the primary cilium only in RPE cells. The authors are correct that how primary cilia in the RPE are affecting RPE function and neural retina health has yet to be studied. Their approach to utilize a conditional knockout mouse model to ablate primary cilia exclusively within the RPE cells is very elegant. However, I do find that their conclusions are a bit heavy handed when discussing the impact RPE cilia defects may play in human ciliopathies. While their work does nicely show that ablating the cilia in RPE cells can lead to underlying retinal defects, it should not be presumed that this insult would be greater than the direct insult of loss of ciliary function within the photoreceptor itself. For instance, the authors make a misleading statement in line 28-29 "Our findings that cilia defects in the RPE precede and contribute to retinal degeneration should be considered when designing treatment strategies for retinal degeneration." Here, precede is not accurate as they cannot discount that in humans the ciliary photoreceptor defect would be driving primary retinal dystrophy and any impact from RPE would be secondary. I also have issue with some of the statements and quantifications made regarding TEM images. Finally, I found the organization of the manuscript cumbersome as the final figures showing RPE morphology and transcriptional profiles were underwhelming compared to the earlier figures showing dysfunction of RPE/retina. I would argue that the transcriptional data set and analysis shown in Figure7-8 should be supplemental information as none of the candidates were validated or followed up in any depth. I feel that reorganizing the manuscript would be beneficial.

*Response: We thank the reviewer for the appreciating our approach and the importance of these investigations. In line with their suggestions, we have thoroughly reorganized the data and figures. We have added additional data analysis and removed much of the transcriptional data. We are optimistic that the adoption of the short report format will enhance the conciseness of the story.*

*We have also dampened down the collusion on the impact of ciliopathies and made it clear that the effects are likely to be less impactful than the loss the ciliary function within the photoreceptor itself. We have toned down the offending sentence and highlighted this more in the discussion. However, we do think that the role of cilia dysfunction is important to acknowledge and want to make sure that this key finding is discernible for the wider field of ophthalmology.*

Major Remarks:

* I am not convinced that the term vacuole is appropriate for what is shown in the TEM images (Figure 4). Vacuoles are defined as fluid-filled spaces surrounded by a membrane that are within a cell. It is not clear that these empty regions are within any cell. They appear to be subretinal gaps between the RPE and photoreceptor outer segments. Considering you already have evidence that there is reduced attachment between RPE and outer segments, I am not surprised that these holes are seen in the TEM images in the Ift20null;Tyrp2-Cre mice, however, referring to them as vacuoles is misleading. It is well documented that fixation artifacts are increased in models of retinal degeneration due to loss of outer segment material and/or photoreceptor cells. There is no evidence that these are cellular vacuoles.
- Additionally, quantification of these vacuoles shown in Figure 4c was not properly performed. The variation between mice must be considered and shown on the graph. The graph must show the #vacuoles counted in each mouse for each genotype. Instead of showing the total # of vacuoles counted.

*Response: Thank you for this observation. We ourselves where not clear how best to describe this phenomenon. The reviewer is quite correct that these are not ‘within’ any cell and as such best not to be described as vacuoles. We now refer to them as ‘subretinal gaps’ between the RPE and photoreceptor outer segments and have highlighted that this might be a consequence of reduced attachment between RPE and outer segments. We have also mentioned that this might be a consequence of fixation artifacts which might be increased in models of retinal degeneration due to loss of outer segment material and/or photoreceptor cells. Finally, we have also added the quantification of number of ‘gaps’ found per section.*

* The accumulated debris in the Ift20null;Tyrp2-Cre mice TEM image is not well labelled (Figure 4d). The red arrow appears to be pointing at the apical RPE.

*Response: This arrow was indeed supposed to be pointing to the apical RPE in order to highlight that the possibly detached microvilli or un-phagocytosed POS are not in connection with the apical RPE. We have tried to make this clearer in the text and have encircled the accumulated debris.*

* Lines 335-337: "Since our Cre driver is already active during embryonic development, we are not able to address the question of what these remaining ciliated RPE cells are doing and how they might be contributing to RPE function post development." - Have you examined the Ift20null;Tyrp2-Cre mice to assess % ciliation in adult RPE? If the 12.5% of ciliated RPE cells at E16.5 all end up keeping their cilium in adult, then there may be no difference between controls. The final % ciliation in adult could be important.

*Response: This is a really important question and something that we are keen to continue exploring. As the reviewer might be aware, assessing ciliation in mature RPE is not trivial. Due to the attachment and intercalation between photoreceptor outer segments and the RPE, teasing these two tissues apart disrupts the apical surface of the RPE, thereby confounding quantification of apical cilia. We have attempted to analyze cilia in six-month-old RPE from both our control and mutant mice but were not able to detect any ciliated structures. This might be due to the processing problems just described, or a true result. For future examinations we want to explore further processing and imaging techniques as well as checking alternative ages.*

* The observations that there are increased macrophages and that RPE cells are growing in area would suggest that the # of RPE cells is reducing. Were RPE cells/area counted?

- More discussion about these results is needed.

*Response: This data has now been moved to the supplement. Detailed single-cell-analysis of aged RPE has not yet been performed. Considering the request to streamline the manuscript this will be saved for a later publication. We have updated the discussion accordingly.*

Minor Remarks:

* Line 82: I believe this has the wrong citation, should be (14) Jonassen JA et al JCB 2008

*Response: Thank you for this observation. The reviewer is correct, and we have now added the correct reference.*

* Line 108: dcERG stands for direct-coupled ERGs

*Response: This has been amended.*

* Line 148-149: Is it accurate to say that "At 1-month of age no significant differences could be observed between Ift20null;Tyrp2-Cre mice compared to controls." When in the figure there is a * above the scotopic a-wave at 1 month.

*Response: This has been amended.*

* Figure 3h - the bar graph is missing x-axis labels. Intuitively, I realize each color is time and assume it corresponds to the times collected for the representative images in 3F, but needs to be clear.

*Response: We have updated this information in the figure.*

* Figure Legend for 3h. "Quantification revealed that the RPE thickness in Ift20null;Tyrp2-Cre mice was significantly lower already at 1-month of age and the subsequent reduction of the retina was accompanied by thinning of the photoreceptor outer segments, while inner segment thickness did not significantly decline until 10-months of age."

- I do not see a graph showing the significant reduction in the RPE layer at 1-month

*Response: This statement has now been removed. In a previous version we had included RPE thickness data, which was removed due to questions about accuracy in the ability to measure RPE thickness from OCT data. This statement has now been removed.*

* Lines 339-341: "What we could show here is that developmental defects in the RPE lead to it a pathological degeneration which precedes retinal degeneration."

- This is a point of discussion but comes across as it was done. Please change the wording to be less predictive and more hypothetical, as it remains a hypothesis. Such as "Using this mouse we could determine whether the developmental defect…"

*Response: We have reworded the discussion and removed this sentence for more clarity.*

*Line 691: Typo microilli… should be microvilli

*Response: This has been amended.*

**Reviewer 3:**

The manuscript by Kretschmer et al is a straightforward and elegant study showing that ablation of primary cilia exclusively in the RPE precedes and contribute to photoreceptor degeneration and visual impairment. RPE dysfunction can lead to retinal degeneration (RD) and blindness; the authors now show the role of cilia in RPE in this process. The assays described in the study are robust, and well controlled. I enjoyed reading the manuscript and support immediate publication of the manuscript. Considering my high level of enthusiasm for the clear and important message conveyed by the paper, I mostly have textual and technical comments that might be considered by the authors before final publication.

*Response: Thank you for these encouraging comments. We appreciate the textual and technical comments and have incorporated them to the best of our abilities.*

My major conceptual comment on the role of cilia impinging on RPE dysfunction involves the most intriguing aspect of this work, i.e., the developmental timing of RPE cilia being affected and the delayed effects on RD. The authors mention transcripts related to epithelial homeostasis, the visual cycle and phagocytosis in this process. Especially, EMT in mutated RPEs is discussed as a mechanism for RD. This aspect of the manuscript might be made stronger with further corroboration of the RNASeq data with immunofluorescence and/or immunoblotting of the affected proteins and by including any timeline for such effects.

*Response: We are pleased to see that the reviewer is as intrigued as we are regarding the transcriptional data. In light of the fact that we have been asked to reduce the volume of this manuscript, we have now moved the majority of data to the supplement and will focus on expanding these findings in further investigations.*

My major technical comment is regarding data presentation by the authors. Please mention n in all figs, how many experiments or animals tested etc. Show individual data points for counts in graphs. Similarly, please mention SD, SEM, mean/median, box-whisker plots etc in data representation in relevant figures.

*Response: This has now been added to all the figure legends.*

Other comments:

1. Lines 79-80: it's unclear how patchy Cre activity was assessed.

*Response: This statement refers to the Davis et al., 2009 paper which describes the generation and characterization of this Cre Driver. We have now added the reference directly at the end of the sentence so that this more evident.*

2. Lines 86-87: convoluted writing

*Response: We have rewritten these two sentences for clarity.*

3. Line 90: Change to ARl13B

*Response: This has been amended.*

4. Lines 130-133: what age photoreceptors and RPE used?

*Response: We used P16 and 3-month-old mice and determined the adhesion between the photoreceptor outer segments and the RPE respectively. We have amended this sentence to make that clearer.*

5. Lines 179-182: Authors suggest such vacuoles in RPE not observed before. They do see such structures in control, are these related to the Cre?

*Response: These structures were only rarely observed in the control. As suggested by one of the other reviewers we are no longer referring to these structures as vacuoles but as subretinal gaps between the RPE and photoreceptor outer segments. Fixation artifacts can be increased in models of retinal degeneration due to loss of outer segment material and/or photoreceptor cells. Since we already showed reduced attachment between RPE and photoreceptor outer segments, these abnormalities might be increased upon tissue preparation in the mutants. Nonetheless, they highlight a difference in tissue integrity. To quantify the holes, we used semithin sections generated during the processing for transmission electron microscopy. We have now plotted the data from the holes as number of holes per semithin section. This shows how rarely these events were detected in the control despite being Cre positive.*

6. Lines 198-99: what is meant by depth?

*Response: With this we were referring to the distance between the apical RPE membrane and end of the POS. We have now amended the text accordingly.*

7. Lines 387-390: Not sure if authors' previous paper on Bbs8 ko mentions about EMT changes in the ko RPEs.

*Response: It does indeed. In Schneider et al., 2021 - Figure 8A-E we show that loss of Bbs8 induces EMT- like traits in the RPE.*

8. Please mention methods for melanin quantification in Fig 2.

*Response: The methods have now been updated.*

9. Fig 3h. Please clarify how many controls tested in addition to mutants.

*Response: We have now added n number to all figure legends in the manuscript.*

**Reviewer 4:**

The manuscript entitled "Ablation of primary cilia in the RPE leads to retinal degeneration" by Kretschmer and Schneider et al., addresses the intriguing question of what is the role of primary cilia in the RPE cell layer. Although the biogenesis and maintenance of retinal photoreceptor cilia has been investigated in retinal degeneration by numerous studies, the involvement of the cilia made by the RPE layer on retinal degeneration and the underlying disease mechanisms has not been addressed adequately and warrants the current study at hand. Overall the study design and the data presented are of good quality. The authors describe the use of an in-vivo model system to investigate the role of primary cilia of RPE cells utilizing Tyrp2-Cre to delete Ift20. The authors show this leads to retinal degeneration over time and blindness in the conditional KO mice and performs transcriptomic analysis to shed insight into the underlying disease mechanism.

The major caveat of the study however is that it is very descriptive in nature and provides very little mechanistic data on the exact molecular machinery downstream of cilia loss in the RPE leading to retinal degeneration. A revised version of the manuscript addressing the below issues should be considered for publication.

*Response: We are glad that the reviewer could appreciate the intriguing nature of our work and the importance of examining the role of cilia in the RPE for retinal disease mechanisms. In response to the editor's directive, we have now reformatted the manuscript in the form of a short report and look forward to addressing more mechanistic questions in later studies.*

1.) A cilium length quantitation should be added to figure panel 1d.

*Response: This was a very useful suggestion and these data have now been added (see new Figure 1c). Interestingly, of the few cilia remaining in the mutant they appeared longer, again suggesting a defect in ciliary trafficking and length regulation. This has been added to the results.*

2.) Figure panel 1F should also show the Tyrp2-Cre only group as a control.

*Response: This data has now been added.*

3.) It is difficult to gauge what percentage of RPE cells express Tyrp2-Cre by the images shown in figure panel 1h. This is an important question that needs to be addressed to evaluate if the phenotypes observed are resultant by a partial loss-of function. The number of red cells in comparison to the black RPE layer in the bright field image shown on the right seems quite low.

*The Cre driver used in this study has undergone comprehensive characterization by the Ashery-Padan lab, who generated this particular mouse line (Davis et al., 2009). The experiment conducted to demonstrate the loss of IFT20 expression and ciliation is more indicative of active Cre expression. The primary objective of this figure was to establish the specificity of the Cre driver, specifically its restriction to the RPE layer rather than other layers of the retina. In the interest of streamlining the manuscript, we have relocated these data to the supplementary section.*

4.) Figure 2 panel F genotype information is mislabeled and the null group should be identified correctly. There seems to be bleed-through between the DAPI and green channels shown and the authors should utilize arrowheads to identify what was quantified.

*Response: As noted by the reviewer, we have observed that upon immunofluorescent assays in primary cultures there are instances where nuclei also exhibit weak fluorescence in the green channel. However, our quantification software effectively eliminates faint nuclei-associated green signals by applying specific size and brightness criteria. Consequently, we exclusively quantified the intense FITC signals related to the POS in these experiments. To address potential confusion caused by annotating all POS-related FITC signals with arrows in the image, we have incorporated this clarification into the methods section.*

5.) The corresponding OCT images for the control group should be shown to each time point instead of the one-month time point as shown in Figure 3f.

*Response: This has now been added. Considering space limitations, we have kept the original in the figure and added the version suggested by the reviewer in the supplement.*

6.) Please show equal magnification images of the cut outs in figure 4a. Please show higher magnification images for the Cre only control group and utilize arrows to indicate the vacuoles described.

*Response: This has now been amended.*

7.) Can the authors comment on the size of the vacuoles observed and possibly show quantitative data on vacuole size distribution in Figure 4C?

*Response: As suggested by one of the other reviewers we are no longer referring to these structures as vacuoles but as subretinal gaps between the RPE and photoreceptor outer segments. Fixation artifacts can be increased in models of retinal degeneration due to loss of outer segment material and/or photoreceptor cells. Since we already showed reduced attachment between RPE and photoreceptor outer segments, these abnormalities might be increased upon tissue preparation in the mutants. Nonetheless, they highlight a difference in tissue integrity. To quantify the holes, we used semithin sections generated during the processing for transmission electron microscopy. We have now plotted the data from the holes as number of holes per semithin section.*

8.) The Bruch's membrane text should not cover the entire membrane in the figure shown hindering its visualization. Maybe utilize BM abbreviation to mark the layer in figure 4e?

*Response: This has now been amended.*

9.) Please utilize arrowheads and potentially higher magnification images to point out what is described as "discontinuous Bruch's membrane" in figure 4j.

*Response: We have now added red brackets to highlight the discontinuous membrane in mutant RPE.*

10.) It is difficult to make any conclusions based on the data shown in figure 6a-c or its significance to the study. Can the authors present the cell size/area data comparing number of cells with different binned sizes at each age? Do the nulls have significantly fewer cells to accommodate a larger area if there is no difference in cell death?

*Response: We have now displayed the cell area data binned at intervals of 50μm^2^ (Figure 2c). Interestingly the binned profiles look remarkably similar between the control and the mutant across all three ages examined. Suggesting that there are no mayor differences in cell size ratios, despite a slight increase in total cell size in the two older categories.*

11.) Can the authors provide data on if the retinal vasculature was developed normally in the nulls? Are there any differences in the retinal vasculature over time by the loss of RPE primary cilia?

Response: We appreciate the suggestion and acknowledge the value of exploring this in the future. However, considering the current limitations such as time constraints, the necessity to modify our animal protocol, and the additional request to transform this manuscript into a short report, we have decided to reserve this experiment for future analyses.
