# Supplementary material for: Deletion of IFT20 exclusively in the RPE ablates primary cilia and leads to retinal degeneration
Source: PLoS Biol. 2023 Dec 4;21(12):e3002402. doi: 10.1371/journal.pbio.3002402 (PMC10721183; doi:10.1371/journal.pbio.3002402)
Supplement: Attachment — Submitted filename: New Response to Reviewers Comments.docx [file pbio.3002402.s014.docx]

**Response to Reviewers' Comments**

Rev. 1:

Previous reviewer’s comments:

The manuscript by Kretschmer et al is an interesting study regarding the importance of IFT20 in RPE cells to retinal health. The RPE specific knockout of IFT20, a gene known to be crucial for the maintenance of primary cilia, results in a slow, progressive retinal degeneration associated with defects in RPE cells. The data presented are extensive and mostly of high quality. This study does a nice job in quantification of observed phenotypes. The major fault of the paper is that the phenotype induced by knockout of IFT20 occurs throughout the adult life of the mouse (very slow), when few, if any, RPE cells have a primary cilia. How could the effects of IFT20 knockout be exerted through ciliary defects if RPE do not have cilia to begin with? This point is essentially not addressed. Further, the potential non-ciliary functions of IFT20 are not mentioned or explored. This study should be revised to address these issues and present the data about the phenotype associated with IFT20 deletion from the RPE cells, rather than presume that the effects are through cilia ablation.

AU Response:

We appreciate time and effort that this reviewer took to review our manuscript and are grateful that they acknowledged the quality of the data. Previous reports by ourselves and others had shown that cilia in the RPE are required for development and maturation of the RPE, after which the cilium retracts. In this report we wanted to highlight that this has long lasting consequences, ultimately leading to retinal degeneration. We have now expanded this point in the discussion to make this clearer. We have also updated the discussion to explore the possibility of non-ciliary functions of IFT20. In response to the editor's request, we have thoroughly reorganized the data and figures in this manuscript. We are optimistic that the adoption of the short report format will enhance the conciseness of the story.

- New reviewers’ comments:

The switch to a short report format greatly improves the manuscript by getting the message across more concisely. However, there remains the single most major issue, which has not been addressed adequately in the revision. My principal concern remains that the manuscript still makes the unsubstantiated claim that cilia loss is what causes retinal degeneration, when in fact, this has not been shown. What has been shown, is that loss of IFT20 in the RPE causes retinal degeneration. The most critical place that the authors must correct these claims is in the Title and Abstract.

***New response: Thank you for commenting on the improvement of the manuscript. We have worked hard to correct these claims throughout the text and have even mentioned the prospect of non-ciliary functions in the abstract. We have also added some additional points in the discussion. For more details see response to comments below.***

Previous reviewers’ comments

11. Line 346 - The correct point brought up in Discussion that ciliary proteins may have non-ciliary roles is essentially not addressed in this paper. The disease observed by the authors with IFT20 KO RPE is very slow and occurs over a timeframe when the vast majority of RPE do not have cilia. How could a ciliary defect cause this long-term pathology in cells that lost their cilia during development? Specific non-ciliary roles of IFT20 are not discussed, despite a seemingly abundance of literature on the topic (see PMID: 33072760 for recent review). The concept that IFT20 is doing something important in fully developed RPE that lack cilia should be strongly considered/discussed/tested. This concern is highlighted by the fact that the title of the paper starts with "Ablation of primary cilia…" when it should begin with "Knockout of IFT20…".

AU Response:

We have expanded this point in the discussion and make it clear that non-ciliary functions of IFT20 might also be playing a role here. It is plausible that we are witnessing a compounded effect involving both developmental defects leading to immature/pathogenic RPE and subsequent effects resulting from the loss of IFT20 in adulthood. This effect may arise from the remaining few ciliated cells or from non-ciliary functions of IFT20. To investigate this matter further, we intend to employ inducible IFT20-RPE Cre knockout models in future studies.

- New reviewers’ comments:

I’m happy that you agree that it is still uncertain whether the observed phenotype is due to ciliary loss, or through a different, non-ciliary mechanism. I like your idea to use an inducible IFT20-RPE Cre knockout model to address this question in future studies. However, I am confused why the present manuscript still makes the claim that ciliary loss is causing the effects when, at this point, it is still unknown whether ciliary loss or non-ciliary functions of IFT20 are causing the effects. This unproven claim needs to be corrected in the title, abstract, Figure 4 title, section titles and elsewhere throughout the text.

***New response: We appreciate these concerns and have amended the text in several ways. We changed the title and corrected this statement throughout the text. We also expanded the section of discussion regarding alternative functions of IFT20.***

***We also had a long discussion with Greg Pazour (University of Massachusetts Medical School), arguably the worlds expert on IFT20 and related cilia mutant models. We have put together four arguments as to why we believe that the phenotype we see is largely in part due to loss of cilia. These arguments have been described in a new paragraph in the discussion.***

- ***In general, all IFT20 phenotype models correlate strongly with other cilia mutants. In particular with IFT74 and IFT88, which are also components of the IFTB trafficking complex.***
- ***Numerous differentially regulated transcripts that overlap with the differentially regulated biological processes observed in the Bbs8 cilia mutant knockout RPE were also detected by our RNA sequencing analysis.***
- ***IFT20 is not found in any organisms without cilia, suggesting that its function is primarily related to the cilium. In particular since IFT20 is a component of the IFTB complex, the most ancestral ciliary trafficking com-plex.***
- ***Despite many studies showing IFTB proteins involved in cell division, our phenotype was not compatible with defects in cell division and spindle orientation. Defects of which would have been evident in the morphological patterning.***

AU response:

Additionally, we have emphasized that IFT20 remains one of the most appropriate models for investigating the implications of ciliary loss. This is because the loss of other intraflagellar transport proteins (such as IFT88) still allows for the presence of a ciliary stump above the transition zone. Given that IFT20 is essential for ciliary vesicle trafficking to the basal body, its loss generates one of the most comprehensive models for ciliary ablation.

We have also revised the title to reflect the loss of IFT20. However, we find it crucial to emphasize the loss of cilia for readers who may not be familiar with ciliary biology in order to ensure comprehensibility of the article.

- New reviewer’s comments:

The title was changed from: “Ablation of primary cilia exclusively in the RPE leads to retinal degeneration and visual impairment.” To: “Ablation of primary cilia, via deletion of IFT20, exclusively in the RPE leads to retinal degeneration.”

The subject and verb of both sentences does not change, and therefore, both sentences claim “Ablation of primary cilia … leads to retinal degeneration”, which is not shown in this manuscript. A couple examples of appropriate titles would include:

“Deletion of IFT20 exclusively in the RPE leads to retinal degeneration”

“Deletion of IFT20 exclusively in the RPE ablates primary cilia and leads to retinal degeneration”

This same logic in correcting the title, needs to be applied throughout the text and especially in the abstract.

***New response: Thank you for these suggestions. We have decided to go with the second suggestion. And have also updated the text accordingly.***

Rev. 2:

The revised manuscript by Kretschmer et al. is better organized and well-written. The authors made major changes to the manuscript in line with the reviewer and editor's comments. I have no major points that need to be addressed and only a few minor comments.

Minor Comments:

DEG is not a commonly used abbreviation and should not be used. Please spell out differentially expressed genes.

***New response: This has now been amended.***

A description of the different stages of RPE maturation at P11, P29 and 3-month should be provided early, as these ages are highlighted as being important on line 202-205, but not described. Line 202-205 - "Although all three time points reflect different stages of RPE maturation and pathogenic processes, it is still interesting to compare differentially expressed transcripts common across all three data points, as these might reflect possible underlying mechanisms (Supplementary Table 4): ”

***New response: This was a very good idea and we have now included a justification for using these times points in the results were we first show data at P11, P29 and 3 months.***

"Typo on line 154 - "mayor" should be "major"

***New response: This has now been amended.***

Line 277 has a reference formatting error

***New response: This has now been amended.***
